# Giant Seebeck effect in Ge-doped SnSe

**DOI:** 10.1038/srep26774

**Published:** 2016-06-02

**Authors:** M. Gharsallah, F. Serrano-Sánchez, N. M. Nemes, F. J. Mompeán, J. L. Martínez, M. T. Fernández-Díaz, F. Elhalouani, J. A. Alonso

**Affiliations:** 1 Instituto de Ciencia de Materiales de Madrid, C.S.I.C., Cantoblanco, E-28049 Madrid, Spain; 2 Sfax University, National School of Engineers, B. P. W 3038, Tunisia; 3 Institut Laue-Langevin, B.P. 156, F-38042 Grenoble Cedex 9, France

## Abstract

Thermoelectric materials may contribute in the near future as new alternative sources of sustainable energy. Unprecedented thermoelectric properties in p-type SnSe single crystals have been recently reported, accompanied by extremely low thermal conductivity in polycrystalline samples. In order to enhance thermoelectric efficiency through proper tuning of this material we report a full structural characterization and evaluation of the thermoelectric properties of novel Ge-doped SnSe prepared by a straightforward arc-melting method, which yields nanostructured polycrystalline samples. Ge does not dope the system in the sense of donating carriers, yet the electrical properties show a semiconductor behavior with resistivity values higher than that of the parent compound, as a consequence of nanostructuration, whereas the Seebeck coefficient is higher and thermal conductivity lower, favorable to a better ZT figure of merit.

Thermoelectric effect allows the direct conversion of waste heat to useful electrical energy. Thermoelectric devices may be employed in electronic refrigeration and power generation; thus the main aim in this area is to identify effective, environmentally harmless and low-cost solid thermoelectric materials. The great potential of thermoelectric generators has motivated the search for novel materials with an improved performance, evaluated with the figure of merit ZT, defined as ZT = S^2^σT/κ, where S is the Seebeck coefficient, σ is the electrical conductivity, κ is the thermal conductivity and T is the absolute temperature. The figure of merit determines the theoretical fraction of the Carnot efficiency that can be reached by a thermoelectric material. As the transport properties are interdependent, they need to be optimized to get the best ZT. Although the efficiency of thermoelectric generators is rather low, the advantages, such as compactness, absence of mobile parts, quiet operation, and reliability, allow for a wide range of applications^1^,^2^,^3^,^4^,^5^,^6^.

Many different approaches have been undertaken to improve the physical parameters involved in ZT since theoretical predictions proposed that nanostructuration could lead to a great enhancement in thermoelectric efficiency^7^. Complex bulk materials in which high thermoelectric efficiency may be obtained have been investigated because of their inherent low thermal conductivity and high thermopower such as Zintl phases^8^, skutterudites^9^ and clathrates^10^. Only in compounds possessing band structure complexity, high degree of degeneracy, several scattering mechanisms and co-existing bonding types, can these conditions be achieved^11^.

Recently, a new material has drawn the attention of the thermoelectric scientific community, with the description of a record-high ZT value of 2.6 at 923 K in single crystalline SnSe semiconductor^12^. In fact, this compound had been known for a long time but its thermoelectric parameters were evaluated as poor^13^,^14^. Indeed, the unusually high values reported for single-crystalline SnSe correspond to the threshold temperature of a structural phase transition at 820 K from the low-temperature structure, defined in the *Pnma* space group, and consisting of corrugated layers of a NaCl like arrangement of SnSe_3_ (and SeSn_3_) units, to a *Cmcm* structure which is prone to decompose by Se evaporation^15^,^16^.

In polycrystalline specimens, the reported properties for single crystal materials have not been reproduced^16^, where ZT values reach 0.5 at 820 K. Also, different chemical substitutions in this compound in both the Sn and Se sublattices have led to some improvement of certain parameters^17^,^18^. The fact that SnSe is a semiconductor with a low intrinsic defect concentration drove some authors to increase the concentration of extra free carriers, for instance by substituting Ag for Sn. In Ag_0.01_Sn_0.99_Se, ZT was found to be 0.6 at 750 K^18^. By using Na as an effective acceptor an ultrahigh power factor was achieved for single-crystal SnSe in a broad temperature range^19^,^20^.

However this material presents some disadvantages that could impede its application i) SnSe is difficult to prepare in single crystalline form, and hard to handle, as it is prone to cleave^12^,^21^, ii) the preparation described for polycrystalline samples requires long annealing times followed by spark-plasma sintering processes, leading to inhomogeneous phases where the segregation of Sn is frequently observed^14^ and which is also difficult to scale-up, iii) the defects introduced by the synthesis methods and minor impurities have a strong impact on the number of carriers and hence on the Seebeck coefficient and electronic conductivity, leading to a dispersion of the reported data in the literature^18^.

In our recent work on polycrystalline SnSe samples synthesized by arc-melting^22^ we found spontaneous nanostructuration resulting in a low thermal conductivity, while maintaining the high Seebeck coefficient. This encouraged us to further improve these results seeking enhanced thermoelectric efficiency by the doping of SnSe.

In this report we describe a fast and straightforward preparation procedure of compact pellets of Sn_1−x_Ge_x_Se, by arc melting, leading to highly oriented polycrystalline specimens where Ge inclusion into the structure is confirmed by neutron powder diffraction (NPD) studies. When Ge replaces Sn, no significant changes in the charge distribution are expected, since these two elements are in the same column of the periodic table. This dopant may nevertheless increase the phonon scattering factor due to the smaller volume of Ge and the consequent decrease of the unit-cell dimensions. For theses samples we observe record-high Seebeck coefficients related to the change in band-structure and the band-gap energy. Additionally, we also found extremely low thermal conductivities, probably linked to the large nanostructuration. We give a complete characterization of Sn_1−x_Ge_x_Se specimens prepared by arc-melting, including crystal structure (XRD and NPD) refinement, DFT band-structure calculations and transport properties (Seebeck, electrical and thermal conductivity).

## Results and Discussion

**Figure 1:**
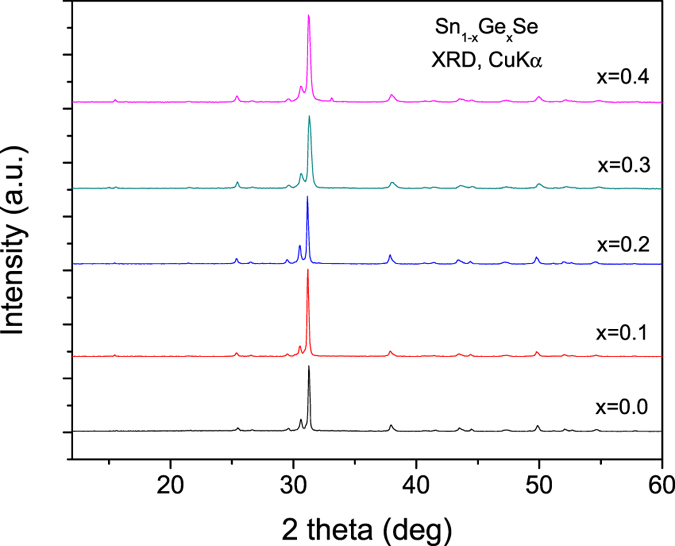
XRD patterns for as-grown Sn_1−x_Ge_x_Se (x = 0, 0.1, 0.2, 0.3, 0.4) intermetallic compounds.

Figure 1 shows the XRD patterns of Sn_1−x_Ge_x_Se samples (x = 0, 0.1, 0.2, 0.3) including that of the parent SnSe compound. All the diagrams correspond to well-crystallized GeSe-like structures with orthorhombic symmetry defined in the space group *Pnma*.

**Figure 2:**
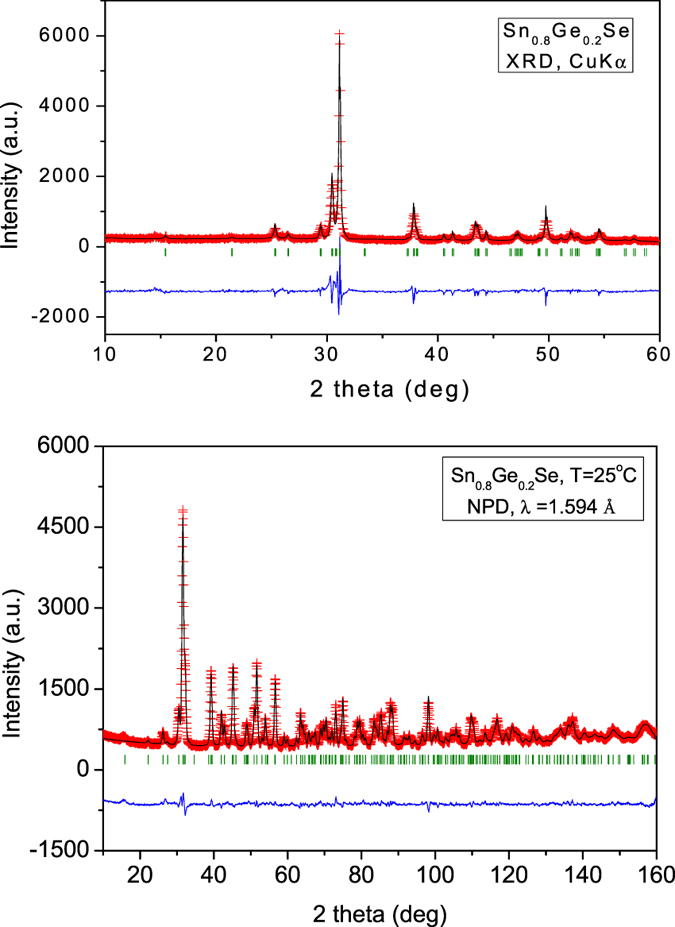
Rietveld plots for Sn_0.8_Ge_0.2_Se from (**a**) XRD data, defined in the space group Pnma. A strong preferred orientation is observed, enhancing [h00] reflections (**b**) NPD data at RT. Observed (crosses), calculated (full line) and difference (at the bottom).

Figure 2a illustrates the Rietveld plot for the x = 0.2 specimen, refined from XRD data. The XRD patterns all exhibit a strong preferred orientation enhancing the (h 0 0) reflections; this effect is minimized in the neutron diffraction pattern in Fig. 2b.

**Figure 3:**
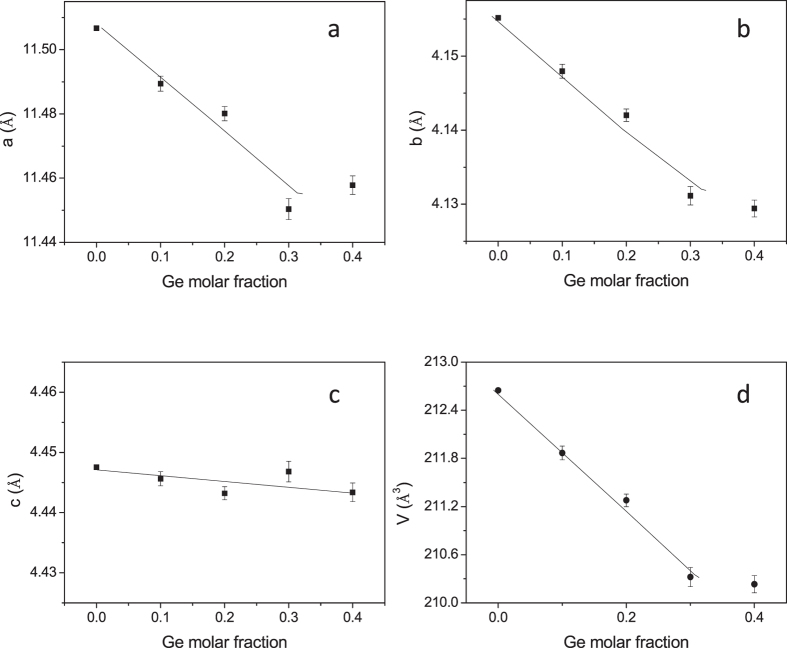
Evolution of the unit-cell parameters of as-grown Sn_1−x_Ge_x_Se (x = 0, 0.1, 0.2, 0.3) (**a**) **a** parameter, (**b**) **b** parameter, (**c**) **c** parameter, (**d**) unit cell volume. The lines are guides for the eye.

Figure 3 displays the variation of the unit-cell parameters and volume with *x* Ge doping. The unit-cell sizes substantially differ from that of the parent SnSe compound (with unit-cell parameters**: a** = 11.5067(1), **b** = 4.1551(1) and **c** = 4.4475(2) Å), showing an overall reduction of a, b and c parameters and unit-cell volume, V, as Ge is introduced into the crystal structure. This reduction happens because the atomic size of Ge is smaller than that of Sn. For instance, for x = 0.2, **a** = 11.4827(8), **b** = 4.1430(3) and **c** = 4.4443(3) Å.

For x = 0.4 the unit-cell parameters and volume do not seem to vary much with respect to the previous composition, therefore we consider x = 0.3 is the maximum Ge content the system is able to accept under the current preparation conditions and thus the x = 0.4 will be excluded from this study.

The investigation of the crystal structure by neutron powder diffraction (NPD) was essential to unveil structural details of the selected composition Sn_0.8_Ge_0.2_Se, since the bulk analysis provided by neutrons is excellent to minimize the preferred orientation effect, given the packing of the ground crystals in vanadium cylinders and the rotation of the sample holder during the experiment. Furthermore, neutrons allow us to explore a much wider range of the reciprocal space, whereas the lack of form factor permits the precise determination the anisotropic displacement factors. The crystal structure refinement from the NPD data at RT was carried out in the SnSe-type model^23^ defined in the orthorhombic *Pnma* space group, with Sn and Ge atoms distributed at random over 4*c* (x 1/4 z) positions and selenium also at 4*c* (x 1/4 z) positions. Table 1 includes the atomic parameters and displacements factors; Table 2 lists the main interatomic distances and angles after the refinement.

**Figure 4:**
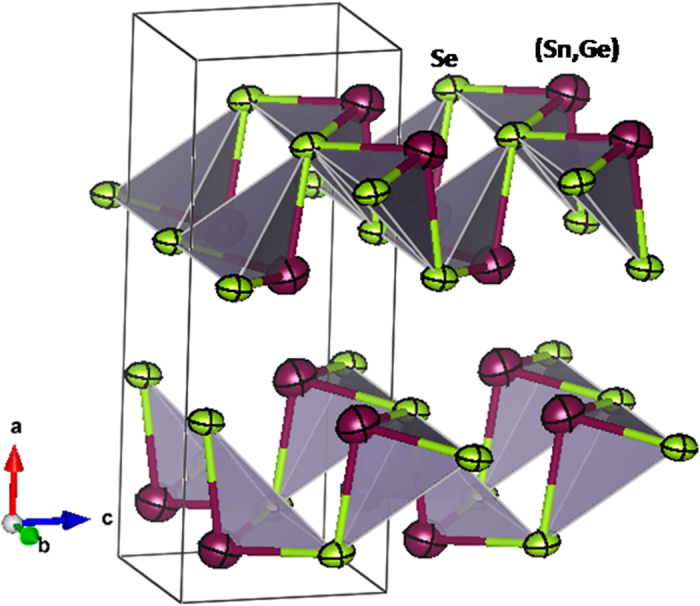
Crystal structure of Sn_0.8_Ge_0.2_Se including 95% probability displacement ellipsoids. The coordination polyhedra (trigonal pyramids) (Sn,Ge)Se_3_ are enhanced.

Trial refinements with Ge placed at the Se positions were unsuccessful, demonstrating that Ge is structurally replacing Sn atoms in the crystal. Figure 2b shows the good agreement between observed and calculated NPD profiles for x = 0.2, with correspondingly good discrepancy factors (R_p_ = 2.47%, R_wp_ = 3.20%, R_exp_ = 1.76%, χ^2^ = 3.29, R_Bragg_ = 3.17%). Figure 4 illustrates the crystal structure of Sn_0.8_Ge_0.2_Se, consisting of puckered layers of (Sn,Ge)Se_3_ polyhedra sharing corners, perpendicular to the **a** unit-cell parameter.

Selenium atoms also form Se(Sn,Ge)_3_ trigonal pyramids; in both cases the lone electron pairs of Sn, Ge and Se are directed to the interlayer space. The comparison of the (Sn,Ge)-Se bond lengths, of 2.745(2) and 2.7897(13) (x2) Å for the x = 0.2 compound, with those observed for pristine SnSe^22^, (2.750(4) and 2.798(2) (x2) Å) shows that the coordination polyhedra become smaller upon Ge introduction, as expected. It is also interesting to consider the interlayer distances, given by Sn-Se bond lengths parallel to the **a** axis, shortening from pure SnSe (3.465(4) Å)^22^ to Sn_0.8_Ge_0.2_Se (3.457(4) Å, Table 2), concomitant with the reduction of the **a** unit-cell parameter. Additionally, the analysis of the neutron data yielded accurate anisotropic displacement factors for all the atoms, displayed in Fig. 4. It is remarkable that the displacement ellipsoids for Se atoms are coplanar with the covalent layers, whereas the vibration direction for (Sn,Ge) atoms is almost perpendicular to the three bonding directions, following the threefold axis of the trigonal pyramid. This feature will be discussed below.

**Figure 5:**
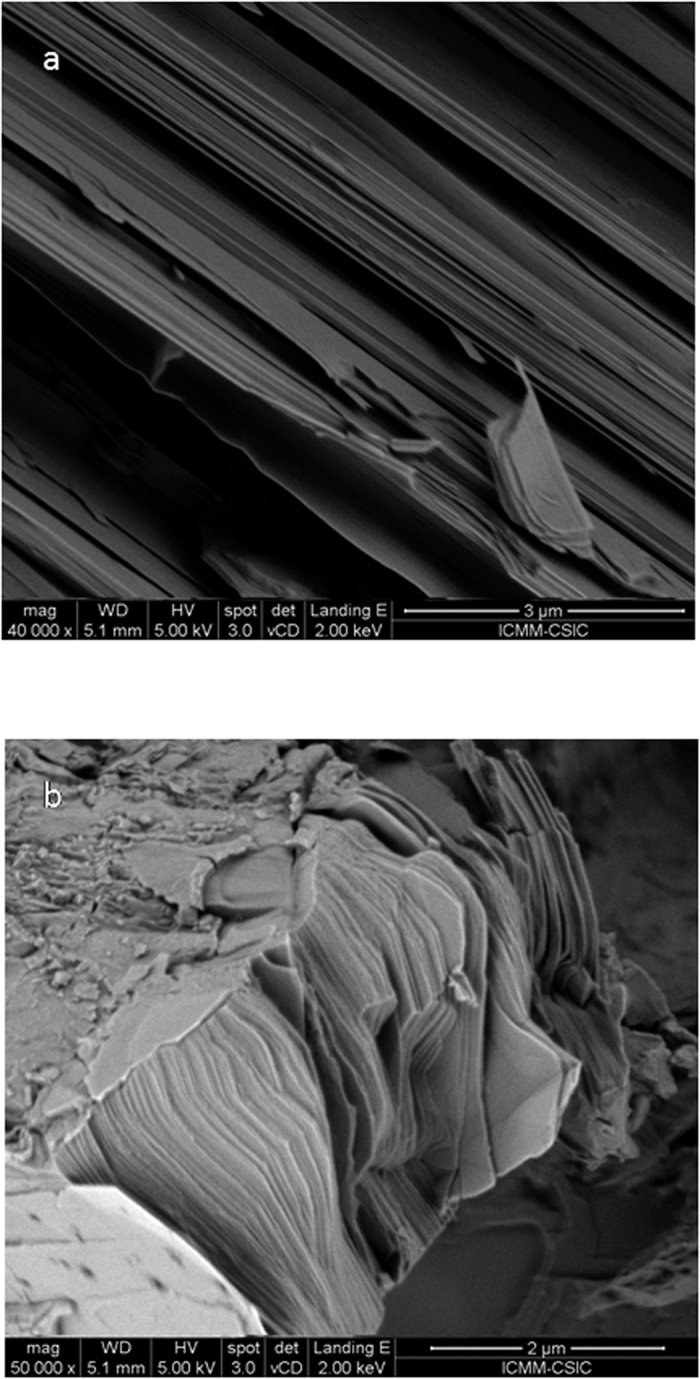
SEM images of as-grown Sn_0.8_Ge_0.2_Se, exhibiting a nanostructure consisting of platelets (perpendicular to [100] direction) for (**a**) x40000 and b) x50000 magnification, showing typical platelet thickness between 20 and 40 nm.

The texture of the as-grown Sn_0.8_Ge_0.2_Se pellets is illustrated in Fig. 5; this is representative of those observed for all the compositions of the Sn_1−x_Ge_x_Se series. The sample is made of piles of stacking sheets parallel to the plane defined by **b** and **c** crystallographic axis, giving the ease of cleavage of this material. The thickness of the individual sheets is well below 0.1 μm (typically 20 to 40 nm). We will show that the thermoelectric properties of this material are strongly influenced by this nanostructuration, involving many surface boundaries that are responsible for scattering of both carriers and phonons. We describe a decrease of the thermal conductivity, which is desirable from the point of view of a thermoelectric material, and as a drawback a deterioration of the electrical conductivity.

### Ab initio calculations

**Figure 6:**
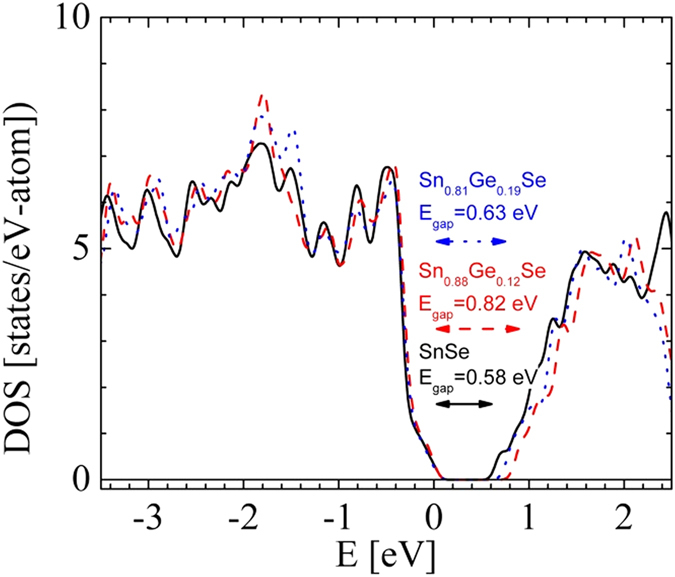
Calculated electronic density of states (DOS) of SnSe (black solid line), Sn_0.88_Ge_0.12_Se (red dashed line) and Sn_0.81_Ge_0.19_Se (blue dotted line).

We performed preliminary GGA DFT calculations with PBE pseudopotentials in CASTEP using the Materials Studio package^24^. We included the experimentally determined unit cells without geometry optimization for each composition. We considered 1*2*2 minimal supercells replacing between 0 and 3 Sn atoms with Ge out of 16. We checked two different configurations for each composition. Figure 6 shows the electronic density of states (DOS) in the valence and conduction bands for various compositions of Sn_1−x_Ge_x_Se for x between 0 and 0.19.

The Fermi-energy is pinned to the top of the valence band for each (semiconducting) composition. For SnSe we find a gap around 0.6 eV, similar to ref. 12 although much smaller than recent calculations^25^,^26^ or the experimental value of 0.923 eV^14^. Nevertheless, the relative changes of the gap between compositions can be considered to be more reliable. Both Ge-doped alloys are predicted to be p-type semiconductors. Although their band structures (not shown) are qualitatively similar to SnSe, the gap of the lighter doped Sn_0.88_Ge_0.12_Se is actually larger by 0.2 eV. This may have implications for its larger observed Seebeck coefficient. The calculated Mulliken charges are between 0.23 and 0.26 for Ge and Sn, mostly taken from the p-levels, whereas around 0.5–0.55 electrons are added to the Se-p levels, and 0.28–0.3 taken from the Se-s levels.

### Transport properties

**Figure 7:**
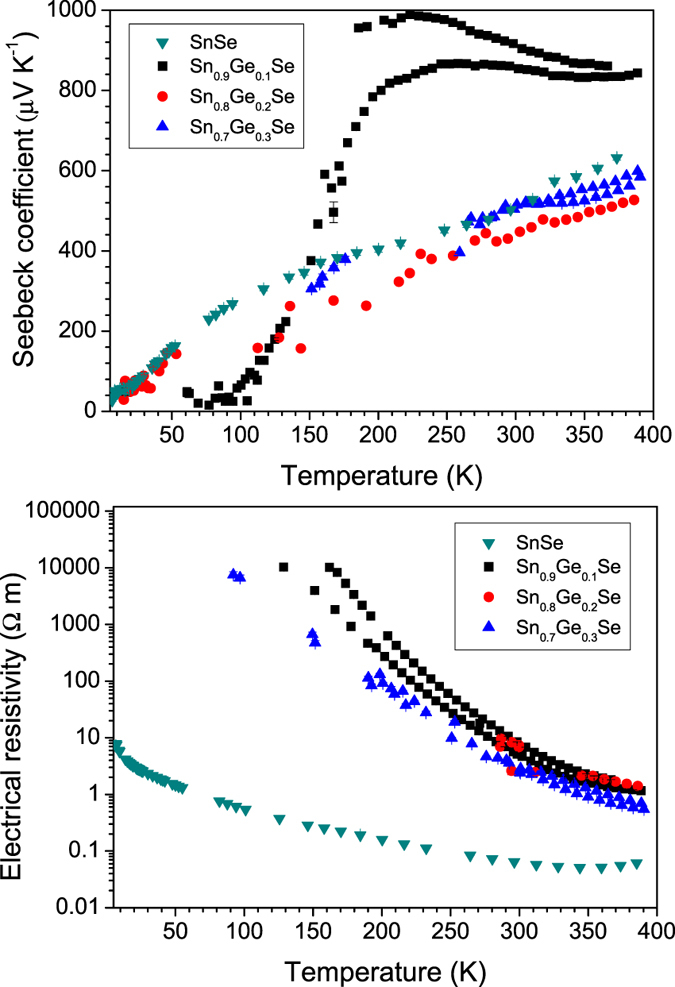
(**a**) Seebeck coefficient vs temperature for Sn_1−x_Ge_x_Se (x = 0.1, 0.2, 0.3) compounds (**b**) Thermal variation of the electrical resistivity, exhibiting characteristic semiconducting behavior. Both panels include the data for SnSe taken from ref. 16. Two independent measurements are shown for x = 0.1 and 0.3. Below around 150–200 K the resistance of the Ge-doped SnSe samples increases beyond the limits (few MΩ) of the electronics used and this influences the Seebeck voltage, too.

In Fig. 7a the Seebeck α coefficient *vs* temperature is shown for all the Ge-doped SnSe stoichiometry. The most remarkable feature is the high value of this coefficient for the Sn_0.9_Ge_0.1_Se compound.

It corresponds to a p-type semiconductor, as found for the parent compound SnSe. We can speculate that the addition of a small percentage of Ge occupying the regular Sn positions may cause lower mobility of the charge carriers due to the smaller size of Ge atoms; besides the larger energy gap found in our theoretical calculations would diminish the number of these thermally excited carriers. This effect is observed in the lowest x = 0.1 substitutional level, where we obtain the largest change in Seebeck coefficient values with respect to pure SnSe. It is noteworthy that the x = 0.1 sample presents, at 258 K, the highest Seebeck coefficient (867 μV K^−1^) reported for the whole series. A second specimen with the same composition reached maximum values close to 1000 μV K^−1^ (Fig. 6a). This is connected to its lowest number of charge carriers, seen in its increased resistivity, as described below. Increasing amounts of Ge reduces again the Seebeck coefficient values close to those of pure SnSe, most likely as a consequence of the contracted band gap. The Seebeck coefficients for x = 0.2, 0.3 are similar to undoped SnSe between 100 and 390 K, reaching a maximum absolute value of 550 μV K^−1^ for x = 0.3. There are no published data on SnSe samples showing similar resistivities to compare with the Seebeck coefficients, even our pure SnSe samples are also considerably more conducting. Zhao *et al.* published results on p-type single crystal SnSe which presents Seebeck coefficient between 500 and 550 μV K^−1^ in the temperature range 300–400 K for all the crystallographic directions. Our Ge doped samples with x = 0.2, 0.3 and pure SnSe samples, prepared by arc-melting, show similar values and behavior, while for x = 0.1 we found greater Seebeck coefficient (above 800 μV K^−1^) that is almost constant in this temperature range^12^.

Figure 7b presents the electrical resistivity for the Sn_1−x_Ge_x_Se series. For all the compounds the resistivity decreases as temperature rises, displaying a semiconducting behavior. For all doping levels there is a noticeable degradation of the electronic conductivity with respect to SnSe (64 mΩ.m at 295 K for the pure sample prepared by arc melting under similar conditions^22^, also included in Fig. 7b for the sake of comparison). Theoretical calculations show that germanium inclusion induces an increased band-gap energy; additionally the reduced size of Ge may cause point defects into the crystal lattice driving a lower mobility of the charge carriers. As the Ge doping level increases, changes in lattice deformation and band-gap energy leads to a reduction of the resistivity, i.e. from about 4.76 Ω.m for x = 0.1 at 300 K down to 2.71 Ω.m for x = 0.3. The lowest resistivity value observed for doped samples (0.696 Ω.m for x = 0.3 at 390 K), is above those described elsewhere for SnSe, perhaps due to an atomic disorder alloying effect. p-type single crystal SnSe data described by Zhao *et al.*^12^ presents an electrical resistivity of 5 mΩ.m to 10 mΩ.m at the temperature range 300–400 K along the **a** crystallographic direction, and 1 mΩ.m to 1.1 mΩ.m within the **bc** plane.; in polycrystalline samples Chen *et al.*^18^ gave 9 mΩ.m and 7 mΩ.m in two different samples and Sassi *et al.*^16^ reported 11 mΩ.m parallel to the pressing direction of the pellet and 5 mΩ.m perpendicular to this direction. This difference may be related to the strong nanostructuration observed for our samples, enhancing the scattering of both phonons and charge carriers, given the large numbers of extended grain boundaries across the layered microstructure described above. Optimization of the electrical conductivity requires further work. The large electrical resistivity in these samples is a consequence, partially, of the low charge concentration, but mostly of the low mobility. Note that at low temperatures, around 150–200 K, the resistivity of some samples increases above the limits of the electronics.

**Figure 8:**
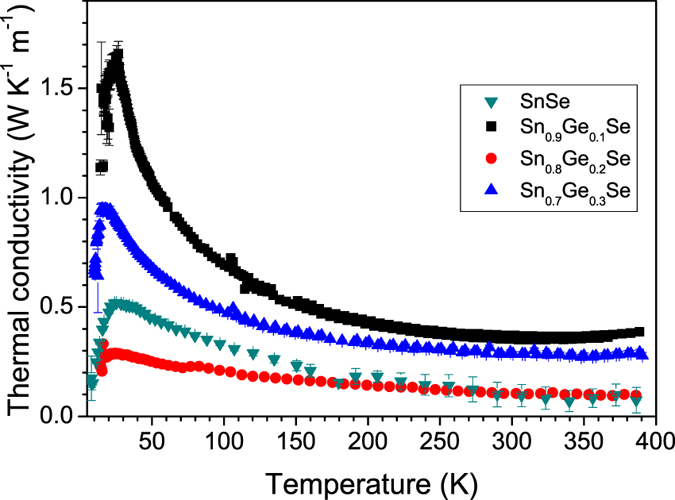
Thermal conductivity vs temperature, for Sn_1−x_Ge_x_Se (x = 0^16^, 0.1, 0.2, 0.3).

The thermal conductivity is presented in Fig. 8. The contribution of the electronic thermal conductivity must be negligible as a consequence of the high electronic resistivity. Thus, these measurements mostly represent the lattice thermal conductivity. For all the doped stoichiometries, the thermal conductivity presents the Umklapp behavior, peaking at low temperatures around 20–30 K, and then it decreases from around 1.2 to 0.2 W m^−1^ K^−1^ over the measurement range. The general trend is a monotonous decrease of the thermal conductivity as temperature increases, with an observed lowest value of 0.23 W m^−1^ K^−1^ at 390 K for the Sn_0.8_Ge_0.2_Se composition. For single-crystal SnSe reported thermal conductivity values in the range 300–400 K are around 0.46–0.37 W m^−1^ K^−1^ along the **a** crystallographic direction and 0.7–0.5 W m^−1^ K^−1^ along the **b** and **c** directions^12^. Radiative correction was used for the higher temperature data.

Intrinsically, this compound has a very low lattice thermal conductivity which is probably a consequence of anhamonicity of the chemical bonds. As an effect of the presence of the lone-electron pairs of both Se^2−^ and Sn^2+^ ions, high Grüneisen parameters and strong phonon-phonon interactions are expected^27^,^28^. In fact, the lone pairs of p-block elements play an important role deforming the lattice vibration, which results in strong anharmonicity. As determined from the neutron study, the atomic vibrations are significantly more anisotropic for Se atoms, carrying two lone electron pairs in the ionic limit; the thermal ellipsoids for Se are contained within the covalent layers since the vibrations are hindered out of the bonding direction by the voluminous electron pairs filling the empty space in between adjacent layers. The significant anisotropy for Se is a symptom of the mentioned anharmonicity. For (Sn,Ge) atoms this effect should be less dramatic since they both carry a single electron pair (in the ionic limit) (Fig. 4).

Finally, the observed nanostructuration in sheets perpendicular to the **a** axis (Fig. 5), spontaneously favoured by the arc-melting technique and most probably by the dramatic quenching conditions undergone by the samples (abruptly cooled from the molten state to the solid ingots in a matter of seconds) seems to be crucial for the exceptionally low thermal conductivities, driven by the many layer-to-layer grain boundaries across the phonon paths. This effect is very favorable for thermoelectric materials, and in the present Ge-doped series it is complemented by the extraordinarily high Seebeck coefficients, reaching 1000 μV K^−1^ for low Ge-doping levels.

## Conclusions

A novel series of Ge-doped SnSe intermetallic compounds has been prepared by a straightforward procedure producing strongly nanostructured samples. Structural characterization shows the decrease in lattice parameters as Ge fraction is increased and an anisotropy and anharmoinicity of the thermal displacements, mostly for Se atoms. Nanostructuration plays an important role as grain boundaries boost the phonon scattering which is the cause of such extremely low thermal conductivity well below the reported values in literature for SnSe. However, these boundaries probably are the main cause for the large values of resistivity which are observed for all compositions, therefore adverse for the thermoelectric efficiency. Also, Ge-doping at low levels greatly affects the Seebeck coefficient values, while at higher Ge molar fraction they exhibit similar values to those of pristine SnSe. Nanostructuration presumably does not play a major role in the enhancement of the Seebeck coefficient as our nanostructured undoped SnSe shows similar Seebeck coefficient to published data on single crystal SnSe. This behavior is anticipated by DOS calculations, where a difference in the band-gap is observed between the doped and the parent compound. We conclude by saying that the strength the arc-melting method lies in the easiness with which modified intermetallic compounds can be synthesized and their properties rapidly assessed.

## Methods

Sn_1−x_Ge_x_Se (x = 0.1, 0.2, 0.3, 0.4) intermetallic alloys were prepared in an Edmund Buhler mini-arc furnace. A mixture of stoichiometric amounts of Sn, Ge and Se powder metals was pelletized under N_2_ atmosphere in a glove box; the pellets were arc-molten under Ar atmosphere in a water-cooled Cu crucible, leading to compact ingots, which were ground to powder for structural characterization, or cut with a diamond saw in bar shaped specimens for transport measurements.

The reaction products were characterized by XRD with Cu K_α_ radiation using a Bruker-AXS D8 diffractometer (40 kV, 30 mA), controlled by DIFFACT^PLUS^ software, in Bragg-Brentano reflection geometry with Cu Kα radiation (λ = 1.5418 Å). A room-temperature NPD pattern was collected for a selected Sn_0.8_Ge_0.2_Se composition, at the D2B diffractometer of the ILL neutron source (Grenoble), with a wavelength λ = 1.594 Å. The sample was packed in a cylindrical vanadium holder (dia. 8 mm), and the counting time was 3 h in the high-intensity mode; the sample holder was rotating during the acquisition time. The refinement of the structure was performed by the Rietveld method and the FULLPROF refinement program^29^.

A Thompson-Cox-Hastings pseudo-Voigt function was chosen to generate the line shape of the diffraction peaks. The coherent scattering lengths for Sn, Ge and Se were, respectively, 6.225, 8.185 and 7.970 fm. A preferred orientation correction was applied, considering platelets perpendicular to the [100] direction. No regions were excluded in the refinement. The following parameters were refined in the final runs: scale factor, background coefficients, zero-point error, pseudo-Voigt corrected for asymmetry parameters, positional coordinates and anisotropic displacements.

Seebeck coefficients, electrical and thermal conductivity, were simultaneously measured in a thermal transport option (TTO) setup within a Physical Properties Measurements System (PPMS) by Quantum Design. The measurements were carried out in the residual vacuum of He atmosphere, under a pressure of 10^−5^ Torr, in the temperature range of 5 to 380 K. The typical size of a Sn_0.8_Ge_0.2_Se pellet was 10 × 3 × 2 mm^3^ with four Cu wires attached with Ag paste (EPO-TEK® H20E). A constant temperature gradient of 3% was applied across the sample during the whole measurement process.

## Additional Information

**How to cite this article**: Gharsallah, M. *et al.* Giant Seebeck effect in Ge-doped SnSe. *Sci. Rep.* **6**, 26774; doi: 10.1038/srep26774 (2016).

## Figures and Tables

**Table 1:** Crystallographic data for Sn_0.8_Ge_0.2_Se from NPD data at RT.

*Crystal data*						
Orthorhombic, *Pnma*	Constant Wavelength Neutron Diffraction radiation, λ = 1.594 Å					
*a* = 11.4827 (8) Å	*T* = 295 K					
*b* = 4.1430 (3) Å	Particle morphology: powder					
*c* = 4.4443 (3) Å	*Z* = 4					
*V* = 211.43 (2) Å^3^	Specimen preparation: arc melting from Sn + Se + Ge					
***Refinement***						
Rietveld	70 parameters					
*R*_*p*_ = 2.47%	*R*_*exp*_ = 1.76%					
*R*_*wp*_ = 3.20%	*R*_*Bragg*_ = 3.17%					
χ^2^ = 3.29	3198 data points					
Profile function: Pseudo-Voigt	Background function: linear interpolation between a set of background points with refinable heights					
Excluded region(s): 1	Preferred orientation correction: Yes					
***Fractional atomic coordinates and isotropic or equivalent isotropic displacement parameters*** (***Å***^**2**^)						
	*x*	*y*	*z*	*U*_iso_*/*U*_*eq*_	Occ. (<1)	
Sn1	0.11835 (14)	0.25000	0.1038 (3)	0.0227 (10)	0.80	
Ge1	0.11835 (14)	0.25000	0.1038 (3)	0.0227 (10)	0.20	
Se1	0.35515 (11)	0.25000	0.0186 (3)	0.0137 (6)		
***Atomic displacement parameters*** (***Å***^**2**^)						
	*U*^11^	*U*^22^	*U*^33^	*U*^12^	*U*^13^	*U*^23^
Sn1	0.0197 (13)	0.0233 (9)	0.0251 (9)	0.00000	−0.0018 (6)	0.00000
Ge1	0.0197 (13)	0.0233 (9)	0.0251 (9)	0.00000	−0.0018 (6)	0.00000
Se1	0.0089 (7)	0.0146 (5)	0.0175 (6)	0.00000	−0.0013 (6)	0.00000

**Table 2:** Main bond distances (Å) and selected angles (°) for Sn_0.8_Ge_0.2_Se from NPD data at RT.

*Intralayer*			
(Sn,Ge)-Se	2.727(7)	Se-(Sn,Ge)-Se	89.0(2) x2
	2.821(5) x2		95.02(1)
		(Sn,Ge)-Se-(Sn,Ge)	100.1(3) x2
			95.02(1)
***Interlayer***			
(Sn,Ge)-Se		3.497(4)	
